# A comparative analysis of semiannual versus annual rituximab maintenance in patients with refractory idiopathic inflammatory myopathies: a retrospective longitudinal real-world study

**DOI:** 10.1590/1806-9282.20260564

**Published:** 2026-10-12

**Authors:** Rodrigo Lorenzetti Serrano, Fernando Henrique Carlos de Souza, Renata Miossi, Marlise Sítima Mendes Simões Faria, Samuel Katsuyuki Shinjo

**Affiliations:** 1 Universidade de São Paulo, Faculdade de Medicina FMUSP, Division of Rheumatology – São Paulo (SP), Brazil.

**Keywords:** Myositis, Idiopathic inflammatory myopathies, Rituximab, Treatment

## Abstract

**OBJECTIVE:**

The aim of this study was to explore the real-world effectiveness of semiannual versus annual rituximab maintenance administration in patients with refractory idiopathic inflammatory myopathies.

**METHODS:**

In this single-center, retrospective longitudinal study (2010–2025), 45 adults with refractory idiopathic inflammatory myopathies received two initial rituximab infusions (1 g on days 0 and 15). Maintenance rituximab was administered every 6 months (semiannual group, n=14) or every 12 months (annual group, n=31). Baseline immunosuppressive therapy was maintained, and glucocorticoid tapering was guided by clinical and laboratory parameters. Outcomes at months 6–12 and 18–24 included the need for rescue therapies: intravenous methylprednisolone (3 g total) and immunoglobulin (2 g/kg), or prednisone dose escalation.

**RESULTS:**

Baseline demographics, refractory idiopathic inflammatory myopathies subtypes, rituximab indications (cutaneous, muscular, articular, and/or pulmonary activity), and peri- rituximab medications (within 2 months before/after infusion) did not differ significantly between groups (p>0.05). No statistically significant differences in the frequency of rescue therapy use at 6–12 and 18–24 months were detected between the two regimens. Intragroup analysis demonstrated significant reductions in relapse rates and rescue therapy requirements over time (p<0.05), suggesting improvement in both cohorts.

**CONCLUSION:**

In this exploratory cohort, no statistically significant differences were detected between semiannual and annual rituximab maintenance regimens with respect to clinical outcomes, although numerically higher relapse rates were observed in the annual group. These findings suggest that extended rituximab intervals may be feasible in resource-limited settings, but further prospective studies are needed to confirm these observations.

## INTRODUCTION

Idiopathic inflammatory myopathies (IIMs), also referred to as systemic autoimmune myopathies, comprise a heterogeneous group of disorders primarily affecting skeletal muscle, though extra-muscular involvement—such as cutaneous, articular, and pulmonary manifestations—is also common^1^. Management typically involves glucocorticoids, intravenous immunoglobulin (IVIG), and various immunosuppressive or immunomodulatory agents, administered either as monotherapy or in combination^2^. In severe or treatment-refractory cases, the use of biologic therapies or small-molecule agents is recommended^3,4^.

Among the biologic agents employed in IIM, rituximab (RTX) has emerged as a key therapeutic option for refractory disease, with favorable clinical outcomes reported across several studies^5-7^. The conventional RTX regimen for IIM involves two 1 g infusions on days 0 and 15, repeated every 6 months, though an alternative lymphoma-based protocol (375 mg/m^2^ weekly for 4 weeks) is also used. Recent studies have explored alternative dosing schedules: Janardana et al.^8^ demonstrated comparable efficacy using 500 mg on days 0 and 15 every 6 months over 12 months of follow-up, whereas Gamba et al.^9^ reported similar outcomes with a single 1 g dose every 6 months during longer-term observation. Ahn et al.^10^ observed a mean response rate of 68.8% over 6 months, despite heterogeneous dosing intervals.

These findings are particularly relevant in public healthcare contexts, where limited availability may preclude adherence to standard dosing intervals. Moreover, the long-term impact of extended RTX dosing schedules remains insufficiently studied.

Accordingly, this study aimed to evaluate disease activity and relapse rates over a 24-month period in patients receiving standard RTX doses either semiannually or annually in a real-world setting.

## PATIENTS AND METHODS

This single-center, retrospective longitudinal study was conducted between 2010 and 2025 and included adult patients diagnosed with refractory idiopathic inflammatory myopathies (rIIM) as follows: dermatomyositis (DM), according to the 2017 EULAR/ACR criteria^11^; clinically amyopathic dermatomyositis (CADM), based on Gerami et al.'s definition^12^; anti-synthetase syndrome (ASyS), according to Connors et al.^13^; and immune-mediated necrotizing myopathy (IMNM), following the criteria by Allenbach et al^14^.

Refractory IIM was defined by inadequate response to glucocorticoids and at least two immunosuppressive agents (e.g., azathioprine, methotrexate, mycophenolate mofetil, cyclosporine, or leflunomide), administered sequentially or concomitantly for ≥3 months^15^.

The study protocol was approved by the local ethics committee, and all participants provided written informed consent.

As exclusion criteria, patients with inclusion body myositis or other non-IIM myopathies, those with severe infections requiring treatment discontinuation, and individuals receiving RTX doses or intervals inconsistent with the study protocol were excluded.

Data collection. Patient data were extracted from electronic medical records using standardized forms, capturing the following variables: demographic: age, sex, ethnicity; clinical: disease duration, muscle strength, dyspnea, arthritis, cutaneous involvement; laboratory data: C-reactive protein (CRP), erythrocyte sedimentation rate (ESR), complete blood count (CBC), creatinine, transaminases, creatine phosphokinase (CK); imaging: chest computed tomography (CT), pulmonary function tests, muscle magnetic resonance imaging (MRI), and joint ultrasound (when indicated); treatment history: previous and current medications, duration of use, and adverse events.

Disease activity was evaluated across four domains:

- Muscular: clinical worsening on Manual Muscle Testing (MMT-8)^16^ or CK elevation above baseline or ≥ three upper limits of normal;
- Pulmonary: new or worsening dyspnea, decline in pulmonary function tests or imaging, excluding infections;
- Cutaneous: physical examination findings consistent with active disease^17^;
- Articular: presence of arthralgia or arthritis, confirmed with imaging if necessary.

Disease flare/relapse was assessed according to the International Myositis Assessment & Clinical Studies Group (IMACS) concept of clinical worsening after a period of disease stability or improvement, involving one or more core disease activity domains^18^. In this study, relapse was pragmatically operationalized as the need for rescue therapy during ongoing RTX treatment, including methylprednisolone pulse therapy (1 g/day for 3 days), intravenous human immunoglobulin (2 g/kg), and/or initiation or escalation of prednisone dosage. To improve consistency, rescue interventions were considered relapse-related only when accompanied by documented clinical and/or laboratory evidence of worsening disease in at least one predefined organ domain (muscular, pulmonary, cutaneous, or articular), after exclusion of alternative causes such as infection or drug toxicity. Nevertheless, this outcome represents real-world physician-driven therapeutic decisions and should be interpreted as a pragmatic clinical marker rather than a validated composite measure of global disease activity.

Timepoints analyzed. Two primary periods were analyzed: peri-RTX (the 2 months before and after initial RTX infusion), and post-RTX (subdivided into months 6–12 and 18–24).

At each timepoint, relapse events and the affected clinical domains were recorded.

RTX regimens. All patients received two 1 g infusions of RTX on days 0 and 15: semiannual group (received repeated doses every 6 months), and annual group (received repeated doses every 12 months, due to limited drug availability).

RTX infusions were withheld in cases of previous adverse reactions, active infections, or hypogammaglobulinemia. Immunosuppressive therapy remained unchanged post-RTX, and glucocorticoids were tapered based on clinical response.

Statistical analysis. Normality was assessed using the Shapiro-Wilk test. Continuous variables are expressed as mean±standard deviation (SD) or median [interquartile range (IQR): 25th–75th], as appropriate; categorical variables are presented as frequency (%). Intergroup comparisons used Student's t-test or Mann-Whitney U test for continuous variables, and chi-square or Fisher's exact test for categorical data. Proportions were analyzed with 95%CI using a binomial distribution. Statistical significance was set at p<0.05.

## RESULTS

Out of an initial cohort of 84 patients, one was excluded due to a severe infection after the first dose, one for receiving a non-standard RTX dose (e.g., other than 1 g on days 0 and 15), six for having received RTX for less than 24 months, and 31 for being treated with administration intervals differing from the semiannual or annual protocols (Figure 1). Consequently, 45 adult patients with rIIM were included in the final analysis: three with CADM, nine with IMNM, 11 with DM, and 22 with ASyS. Among these, 31 patients received RTX annually and 14 semiannually (Table 1). We performed an aggregated, hypothesis-generating analysis. Of the 45 patients, none had hypogammaglobulinemia, infections, hospitalization, infusion reactions, discontinuation, or mortality.

**Figure 1:**
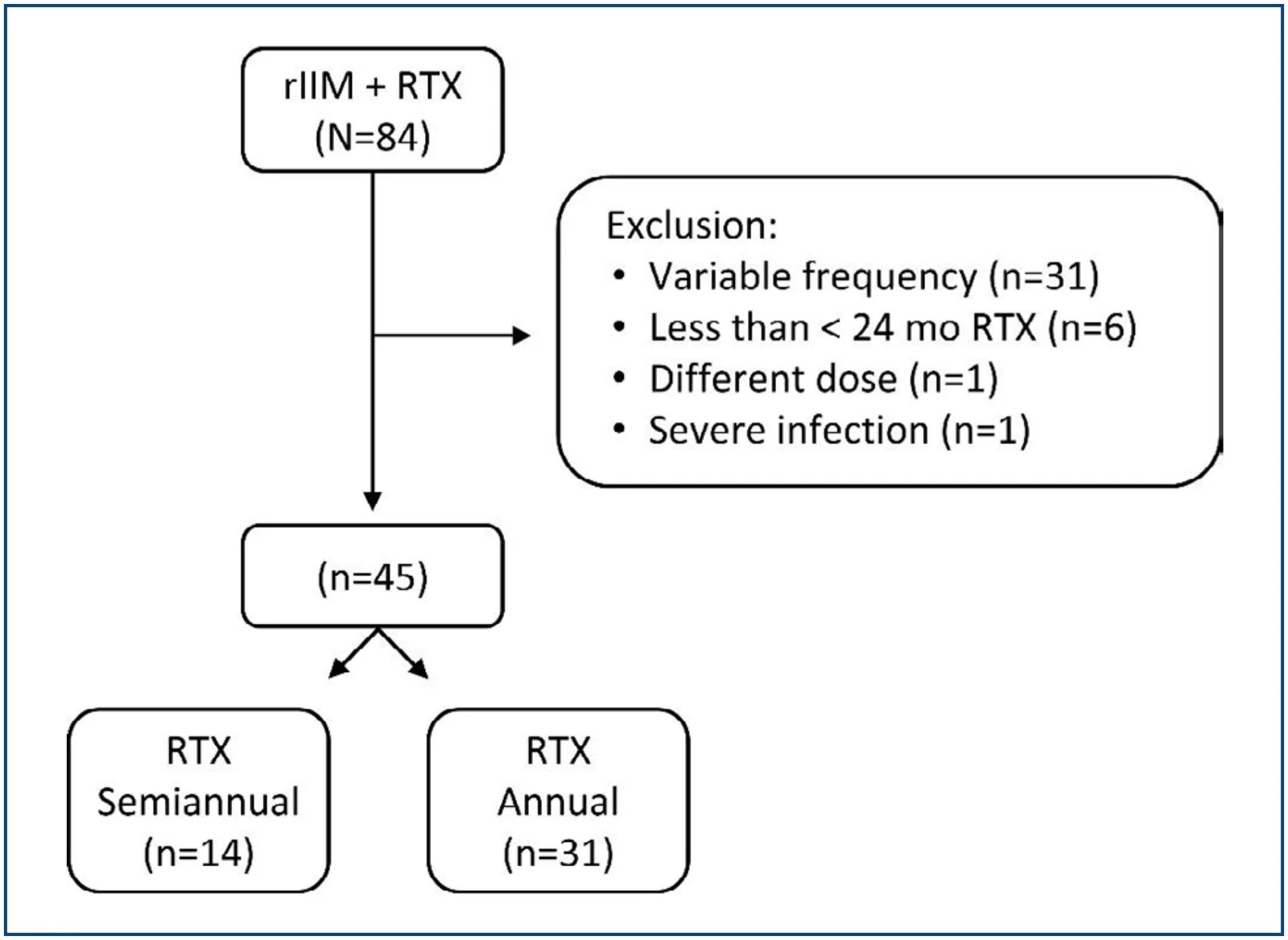
Flowchart of patient selection for the study cohort.

**Table 1:** Demographic and disease characteristics.

		Annual RTX (n=31)	Semiannual RTX (n=14)	p-value	Effect size/odds ratio (95%CI)
Mean age (SD), years		44.6 (±10.8)	46.3 (±8.4)	0.531^a^	d=0.120 (-0.243–0.453)
Sex (female, %)		23 (74.0)	9 (64.0)	0.497^b^	OR 0.626 (0.161–2.433)
Median disease duration (IQR 25th-75th), years		3 (2.0–5.5)	3 (2.0–5.8)	0.872^c^	r_rb_=-0.032 (-0.381–0.324)
IIM subtype (%)					
	DM	7 (22.6)	4 (28.6)	0.717^d^	OR 0.73 (0.14–3.48)
	CADM	2 (6.5)	1 (7.1)	>0.999^d^	OR 0.90 (0.01–15.70)
	AsyS	15 (48.4)	7 (50.0)	>0.999^d^	OR 0.94 (0.23–3.78)
	IMNM	7 (22.6)	2 (14.3)	0.698^d^	OR 1.75 (0.26–18.04)
Antibody (%)					
	Anti-Mi2	1 (3.0)	1 (7.1)		
	Anti-SAE	1 (3.0)	0		
	Anti-Ro52	2 (6.0)	2 (14.2)		
	Anti-NXP2	1 (3.0)	0		
	Anti-TIF1g	1 (3.0)	0		
	Anti-MDA5	0	1 (7.1)		
	Anti-Jo1	8 (25.8)	6 (42.8)		
	Anti-PL7/PL12	5 (16.0)	1 (7.1)		
	Anti-SRP	3 (9.6)	0		
	Anti-HMG-CoA	1 (3.0)	0		
	Anti-PM/Scl	2 (6.0)	0		
Peri-rituximab (%)					
	Methylprednisolone pulse therapy	7 (22.6)	3 (21.4)	>0.999^d^	OR 1.07 (0.19–0.34)
	Intravenous immunoglobulin	4 (12.9)	4 (28.6)	0.231^d^	OR 0.37 (0.05–2.19)
	Prednisone use	26 (83.9)	14 (100)	0.305^d^	OR 0.54 (0.03–5.23)
	Median baseline prednisone dose (IQR 25th-75th) mg	7.5 (5.0–12.5)	12.5 (7.5–20.0)	0.076^c^	r_rb_=0.338 (-0.028–0.624)
Concomitant immunosuppressor (%)					
	0–1	7 (22.5)	4 (28.6)	0.720^d^	OR 1.37 (0.34–5.50)
	≥2	24 (77.4)	10 (71.4)		

ASyS: anti-synthetase syndrome; CADM: clinically amyopathic dermatomyositis; DM: dermatomyositis; IIM: idiopathic inflammatory myopathies; IVIG: intravenous human immunoglobulin; IMNM: immune-mediated necrotizing myopathy; RTX: rituximab; SD: standard deviation; IQR: Interquartile range; CI: confidence interval.

a Student's t-test for independent samples.

b Chi-Square test of independence.

c Mann-Whitney U test.

d Fisher's exact test. Estimates calculated using Haldane-Anscombe correction due to zero cell counts.

The mean age was similar between groups: 44.9 years in the annual RTX group and 46.7 years in the semiannual group (p=0.540). The distribution of rIIM subtypes did not differ significantly between groups: CADM (6.5 vs. 7.1%), IMNM (22.6 vs. 14.3%), DM (22.6 vs. 28.6%), and ASyS (48.4 vs. 50.0%)—all with p>0.05.

The predominant clinical domains prompting RTX initiation—muscular, cutaneous, articular, or pulmonary involvement—were similarly distributed between groups (p>0.05). Regarding median CK levels, there were no differences between groups pre-RTX (167 U/L [137–753] vs. 191.5 U/L [107–1168] for annual and semiannual, respectively; p=0.741), and post-RTX (182 U/L [65–345] vs. 157 [83–260], p=0.750). We also did not find a difference in median MMT-8 score between groups pre-RTX (80 [60–80] vs. 76.5 [69–80], p=0.596), and post-RTX (80 [73–80] vs. 80 [78.5–80], p=0.168).

Peri-RTX treatments also did not differ significantly: methylprednisolone pulses (22.6 vs. 21.4% in the annual and semiannual group, respectively; p>0.999), IVIG (12.9 vs. 28.6%; p=0.231), and prednisone use (83.9 vs. 100%; p=0.305).

During the 6–12-month follow-up period, the need for rescue therapies remained comparable between groups. In the annual RTX group, 22.6% required methylprednisolone, 6.5% received IVIG, and 16.1% required prednisone escalation, compared to 7.1% for each intervention in the semiannual group (p>0.05 for all comparisons). Regarding methylprednisolone, the proportion of patients was numerically higher in the annual group, although the difference was not statistically significant. Muscular relapses occurred in 25.8% of patients in the annual group compared to 7.1% in the semiannual group. Although this numerical difference favored the semiannual regimen, it did not reach statistical significance (p=0.236) (Table 2).

**Table 2:** Rescue therapies and relapse rates at 6–12 and 18–24 months.

			Annual RTX (n=31)	Semiannual RTX (n=14)	p-value	Effect size/odds ratio (95%CI)
Post-rituximab (%)						
	Period: Months 6–12 of follow-up					
		Methylprednisolone pulse therapy	7 (22.6)	1 (7.1)	0.402^d^	OR 3.79 (0.42–34.27)
		IVIG	2 (6.5)	1 (7.1)	>0.999^d^	OR 0.90 (0.07–10.79)
		Prednisone	5 (16.1)	1 (7.1)	0.648^d^	OR 2.50 (0.26–23.67)
Disease activity (relapse)						
	Cutaneous		2 (6.5)	1 (7.1)	>0.999^d^	OR 0.90 (0.07–10.79)
	Muscular		8 (25.8)	1 (7.1)	0.236^d^	OR 4.52 (0.51–40.29)
	Articular		2 (6.5)	0	>0.999^d^	OR 2.46* (0.11–54.60)
	Pulmonary		1 (3.2)	0	>0.999^d^	OR 1.43* (0.05–37.20)
Period: Months 18–24 of follow-up						
	Methylprednisolone pulse therapy		3 (9.7)	0	>0.999^d^	OR 3.56* (0.17–73.71)
	IVIG		2 (6.5)	1 (7.1)	0.488^d^	OR 0.90 (0.07–10.79)
	Prednisone		1 (3.2)	0	>0.999^d^	OR 1.43* (0.05–37.20)
Disease activity (relapse)						
	Cutaneous		0	0	>0.999^d^	OR 0.46* (0.01–24.37)
	Muscular		2 (6.5)	0	>0.999^d^	OR 2.46* (0.11–54.60)
	Articular		1 (3.2)	1 (7.1)	0.262^d^	OR 0.43 (0.03–7.47)
	Pulmonary		1 (3.2)	1 (7.1)	0.262^d^	OR 0.43 (0.03–7.47)

IVIG: intravenous human immunoglobulin; RTX: rituximab; CI: confidence interval.

d Fisher's exact test.

* Estimates calculated using Haldane-Anscombe correction due to zero cell counts.

Between months 18 and 24, a global reduction in rescue interventions was observed. In the annual group, 9.7% received methylprednisolone, 6.5% IVIG, and 3.2% prednisone. In the semiannual group, no patients required methylprednisolone or prednisone, and only 7.1% received IVIG (p>0.05). Relapses were limited to isolated manifestations, with no statistically significant differences.

Intragroup analysis demonstrated a significant reduction in the frequency of rescue therapy use between the 6–12 and 18–24-month follow-up periods within each treatment group, demonstrating a significant reduction over time (p<0.05 for both groups), indicating comparable efficacy and sustained clinical improvement regardless of RTX administration frequency (Table 2).

## DISCUSSION

This study evaluated patients with rIIM treated with RTX administered either semiannually or annually. Over a 24-month follow-up period, no statistically significant differences were observed between the two regimens in relapse rates or the need for rescue therapies. These exploratory findings indicate that extending the RTX dosing interval to 12 months may be a feasible option for selected patients, although this approach is not currently incorporated into standard treatment protocols.

To date, most studies investigating RTX in rIIM have adopted regimens extrapolated from rheumatoid arthritis (1 g on days 0 and 15 every 6 months) or lymphoma (375 mg/m^2^ weekly for 4 weeks, repeated semiannually). Randomized trials such as the RIM Trial, together with multiple observational studies, have supported the efficacy of semiannual RTX administration in reducing disease activity and facilitating corticosteroid tapering^8,9,19^. Standard dosing strategies have been evaluated in cohorts with DM, ASyS, and IMNM^20-23^. However, to our knowledge, no prior study has directly compared extended RTX intervals, such as annual dosing, in this patient population.

Despite the retrospective design, the use of standardized data collection instruments helped minimize information bias. The results provide preliminary evidence that annual RTX administration did not result in statistically significant increases in relapse rates or reliance on adjunctive immunomodulatory therapies. Our study used a pragmatic, treatment-based definition of relapse, which, despite being standardized through objective worsening in predefined disease domains, may carry inherent center-specific prescribing biases.

Temporal analysis revealed a general decline in the need for rescue interventions in both groups, suggesting that sustained B-cell depletion may contribute to long-term disease control regardless of dosing interval. This finding may be particularly relevant in public healthcare systems where logistical and financial constraints can limit access to biologic therapies.

It is important to emphasize that treatment individualization remains essential. Decisions regarding RTX dosing intervals should consider disease subtype, organ involvement, relapse history, and infection risk. The lack of statistically significant differences observed in this study may be attributable to the limited sample size, clinical heterogeneity, and concurrent immunosuppressive therapies. Additionally, the absence of significant changes in creatine kinase levels or MMT-8 scores in either group should be interpreted cautiously, as many patients had subtypes with minimal muscle involvement and received RTX for non-muscular manifestations.

Key limitations include the observational, non-randomized design, small sample size, absence of quality-of-life data, and lack of a validated extramuscular disease activity tool. RTX interval allocation was influenced by drug availability, and baseline treatment patterns suggest that patients in the semiannual group may have had more severe or refractory disease, potentially confounding group comparisons. This factor should be considered when interpreting the numerically lower relapse rates in the semiannual group.

Prospective, controlled trials with larger cohorts are needed to further evaluate the long-term safety and efficacy of RTX across different dosing schedules.

## CONCLUSION

In this real-world study, no statistically significant differences were detected between semiannual and annual RTX regimens in patients with rIIM over 24 months, with respect to relapse rates or need for rescue therapies. While numerically higher relapse rates were observed in the annual group, these did not reach statistical significance. These exploratory findings indicate that annual RTX administration may be a feasible option for selected patients—especially in resource-limited settings—when appropriate clinical monitoring is in place. However, larger prospective randomized studies are needed to confirm these observations and to determine the optimal RTX dosing intervals.

## Data Availability

The datasets generated and/or analyzed during the current study are available from the corresponding author upon reasonable request.
